# Evaluation of a modified meropenem hydrolysis assay on a large cohort of KPC and VIM carbapenemase-producing *Enterobacteriaceae*

**DOI:** 10.1371/journal.pone.0174908

**Published:** 2017-04-06

**Authors:** Adriana Calderaro, Mirko Buttrini, Maddalena Piergianni, Sara Montecchini, Monica Martinelli, Silvia Covan, Giovanna Piccolo, Maria Cristina Medici, Maria Cristina Arcangeletti, Carlo Chezzi, Flora De Conto

**Affiliations:** Unit of Microbiology and Virology, Department of Clinical and Experimental Medicine, University of Parma, Parma, Italy; University Medical Center Groningen, NETHERLANDS

## Abstract

Carbapenem-resistant *Enterobacteriaceae* (CRE) have spread globally and represent a serious and growing threat to public health. The introduction of rapid and sensitive methods for the detection of carbapenemase-producing bacteria is of increasing importance. The carbapenemase production can be detected using non-molecular methods (such as the modified Hodge test, the synergy test, the Carba NP test and the antibiotic hydrolysis assays) and DNA-based methods. In this study, we propose a modified version of a previously described meropenem hydrolysis assay (MHA) by MALDI-TOF MS for the phenotypic detection in 2h of carbapenemase-producing *Enterobacteriaceae*. The MHA was successfully applied to detect carbapenemase activity in 981 well-characterized *Enterobacteriaceae* strains producing KPC or VIM carbapenemases, and in 146 carbapenem fully susceptible strains. This assay, applied also to NDM and OXA-48-producing strains and to CRE with resistance mechanisms other than carbapenemase production, has proved to be able to distinguish between carbapenemase-producing and -nonproducing *Enterobacteriaceae*.

As already stated and as observed in our hands, MHA by MALDI-TOF MS analysis is independent from the type of carbapenemases involved, it is faster and easier to perform/interpret than culture-based methods. On the other hand, it cannot detect other carbapenem resistance mechanisms, such as porin alterations and efflux mechanisms.

## Introduction

Multidrug resistance is now emerging at an alarming rate among a variety of bacterial species, especially Gram-negative rods (GNR) as *Enterobacteriaceae* (EB), *Pseudomonas* spp. and *Acinetobacter* spp., causing both nosocomial and community-acquired infections [1, 2]. One of the most important emerging traits is the resistance to extended-spectrum β–lactams in GNR. Increasing resistance to carbapenems, which are most often the last line of therapy, has recently been reported worldwide [1]. In 2013, the Centers for Disease Control and Prevention assigned the highest threat level to carbapenem-resistant *Enterobacteriaceae* (CRE) and declared that CRE require urgent public health attention [3, 4]. Carbapenemases are classified by Ambler system into class A (mostly *Klebsiella pneumoniae* carbapenemase, KPC), class B or metallo-β-lactamases (mostly Verona Integron-encoded metallo-β-lactamase, VIM, New Delhi metallo-β-lactamase, NDM, and to a lesser extent Imipenem metallo-β-lactamase, IMP) and class D (mostly Oxacillinase-48, OXA-48, and to a lesser extent OXA-162 and OXA-181), based on their aminoacid sequence homology [5, 6]. Acquired KPC, VIM, NDM, and OXA-48 are the prevalent carbapenemases in *Enterobacteriaceae* in Europe [7]. In particular, in Italy, VIM-producing *Enterobacteriaceae* have been detected since the early 2000s, with recent epidemic diffusion of KPC producers and occasional detection of NDM and OXA-48 producers [7]. Moreover, the dramatic increase of carbapenem-resistant *K*. *pneumoniae* has been documented by the European Antimicrobial Resistance Surveillance Network (EARS-Net), which showed that the percentage of invasive isolates of carbapenem-resistant *K*. *pneumoniae* increased to 15% in 2010 to reach 35% in 2013 *vs* 2% before 2009 [8]. The carbapenemase production can be detected through culture-based methods, such as the modified Hodge test (MHT) and the disk diffusion inhibition test (synergy test, ST) used for years [9] or DNA-based methods (e.g., Polymerase Chain Reaction—PCR). The results of culture-based methods are available in 18–24 h, whereas those of PCR assays in few hours but at higher cost [10, 11]. Moreover, PCR, due to its specificity, can only detect known genetic targets encoding carbapenem-resistance genes [12]. More recently other non-molecular tests able to differentiate between carbapenemase-producing and carbapenemase-nonproducing isolates have been proposed: the Carba NP test [13] and the antibiotic hydrolysis assays. Although, the Carba NP test is a rapid biochemical technique, developed in 2012 [13] and recently suggested by Clinical and Laboratory Standards Institute (CLSI) instead of ST as one of the confirmatory tests for suspected carbapenemase production in *Enterobacteriaceae* and other Gram negative rods (such as *Pseudomonas aeruginosa* and *Acinetobacter* spp.), it cannot detect certain carbapenemase types (i.e. OXA-type, chromosomally encoded) [14]. The antibiotic hydrolysis assays could be performed by UV spectrophotometry, a cheap technique allowing to accurately achieve the purpose but with a time-consuming process, which can be shortened by the Matrix-Assisted Laser Desorption/Ionization Time-of-Flight Mass Spectrometry (MALDI-TOF MS) [9]. As a matter of fact MALDI-TOF MS, recently introduced into routine microbiology laboratories for the identification of bacteria and fungi, may be potentially applied in this complex diagnostic process [15].

Here, we propose a modified version of a previously described meropenem hydrolysis assay (MHA) by MALDI-TOF MS for the phenotypic detection of carbapenemase-producing *Enterobacteriaceae* (CPE) strains. To this purpose, a large group of well-characterized KPC and VIM CPE isolates, as well as a negative control group, was used. Moreover, in order to estimate the applicability of MHA on bacterial species with carbapenemase enzyme types different from KPC and VIM, and with resistance mechanisms other than carbapenemase production, this assay was applied to a small selection of reference and clinical strains, including NDM, OXA-48, AmpC, and extended-spectrum β-lactamase (ESBL) producers.

## Materials and methods

### Reference strains

This study included 8 reference strains received from American Type Culture Collection (ATCC), Public Health England’s National Collection of Type Cultures (NCTC) or United Kingdom External Quality Assessment Service (UK NEQAS): 3 *K*. *pneumoniae* carbapenemase-producing strains (ATCC BAA-1705, *bla*_*KPC*_ positive, ATCC BAA-2146, *bla*_*NDM*_ positive, and NCTC 13442, *bla*_*OXA-48*_ positive), 1 *K*. *pneumoniae* carbapenemase-nonproducing strain (ATCC BAA-1706), 2 AmpC strains (*Escherichia coli* NEQAS 2832 and *Enterobacter cloacae* NEQAS 3419) and 2 ESBL strains (*E*. *coli* NEQAS 3253 and *Proteus mirabilis* NEQAS 3341).

### Bacterial isolates

A total of 1185 EB strains isolated from clinical samples and part of the collection of the Bacteriology Section of the Unit of Clinical Microbiology of the University Hospital of Parma (Italy) were included in this study and tested by MHA (Table 1, S1 text).

**Table 1:** Clinical carbapenemase-producing and -nonproducing *Enterobacteriaceae* strains analysed in this study.

Panel	Carbapenemase type/resistance mechanism	Microorganism	No. of strains	Material					
				Rectal swabs	Urine	Respiratory materials*	Blood and other liquids**	Urethral/ vaginal swabs	Other materials***
I	KPC (617)	*K*. *pneumoniae*	603	335	177	49	22	5	15
		*E*. *coli*	7		4	1			2
		*E*. *aerogenes*	6				2		4
		*M*. *morganii*	1			1			
	VIM (364)	*K*. *pneumoniae*	327	163	145	10	4	1	4
		*E*. *cloacae*	17	3	4	3	2	1	4
		*C*. *freundii*	12		10	2			
		*E*. *coli*	4		1			1	2
		*E*. *aerogenes*	2			1	1		
		*K*. *oxytoca*	1		1				
		*S*. *marcescens*	1			1			
II	None (146)	*E*. *coli*	53		45	4			4
		*K*. *pneumoniae*	35		20	8	2	1	4
		*K*. *oxytoca*	14		3	3	3		5
		*E*. *cloacae*	13			6	1	1	5
		*P*. *mirabilis*	8		6				2
		*S*. *marcescens*	7			7			
		*C*. *koseri*	5		2	2			1
		*M*. *morganii*	5		2				3
		*C*. *freundii*	3		1	2			
		*E*. *aerogenes*	2			2			
		*P*. *stuartii*	1		1				
III	NDM (1)	*E*. *coli*	1			1			
	OXA-48 (1)	*K*. *pneumoniae*	1		1				
	Class B^ (6)	*K*. *pneumoniae*	5	4	1				
		*E*. *coli*	1		1				
	AmpC (1)	*E*. *cloacae*	1		1				
	ESBL (36)	*E*. *coli*	21		17		3	1	
		*K*. *pneumoniae*	10	1	6	1		1	1
		*P*. *mirabilis*	3			2			1
		*C*. *freundii*	2			1			1
	Unknown (13)	*E*.*cloacae*	5		4				1
		*K*. *pneumoniae*	4	3	1				
		*E*.*coli*	3		3				
		*E*. *aerogenes*	1		1				
**Total**			1185	509	458	107	40	12	59

*: bronchial aspirate (80), sputum (14), pharyngeal swab (9), bronchoalveolar lavage (2), nasopharyngeal aspirate (1), nasal swab (1).

**: blood (29), peritoneal fluid (5), liquor (2), abdominal drainage fluid (2), bile (1), pleural fluid (1).

***: wound swab (34), bioptic material (10), pus swab (6), peritoneal drainage fluid swab (3), cutaneous swab (2), pus (2), abdominal drainage fluid swab (1), central venous catheter (1).

^ Class B carbapenemase strains other than NDM and VIM.

The first panel (positive control group) included 981 CRE isolates molecularly characterized as KPC producers (617) or VIM producers (364). The second panel (negative control group) contained 146 EB strains fully susceptible to all carbapenems, cephalosporins and not expressing either AmpC, ESBL or any other β-lactamase or carbapenemase enzymes.

The third panel included 58 characterized clinical strains with carbapenemase enzyme types and resistance mechanisms different from those contained in the first panel: 1 NDM, 1 OXA-48, 6 class B other than NDM- and VIM-genotype, 13 carbapenemase-nonproducing CRE, 1 AmpC and 36 ESBL strains.

All strains were identified at species level by using MALDI-TOF MS (score value > 2.0) (Bruker Daltonics, Bremen, Germany) and submitted to the antimicrobial susceptibility testing (AST) with the BD Phoenix system using the Gram-negative NMIC/ID88 or NMIC/ID94 Combo Panels (Becton Dickinson, Sparks, MD, USA) including the biochemical identification in order to confirm the purity of each strain, according to the manufacturer’s instructions. AST was interpreted according to MIC breakpoint criteria of the CLSI on 2014 [16]. The ESBL and AmpC phenotypes were determined by the automated microbiology system (BD Phoenix). All CRE strains were submitted to the phenotypical analysis performed by MHT [17] and disk diffusion inhibition test (ST) (KPC+MBL Confirm ID Kit, Rosco Diagnostica, Taastrup, Denmark), according to the manufacturer’s instructions, and to the genotypical characterization by 2 molecular methods which succeeded during the study: the first method detected *bla*_*KPC*_, *bla*_*NDM*_, and *bla*_*VIM*_, as previously described [18, 19], and the second one also *bla*_*OXA-48*_ (S2 text).

The samples analysed in this study were sent to the University Hospital of Parma for routine diagnostic purposes, and the laboratory diagnosis results were reported in the medical records of the patients as answer to a clinical suspicion or to active surveillance; ethical approval at the University Hospital of Parma is required only in cases in which the clinical samples are to be used for applications other than diagnosis. Anonymization of the strains was also done in this study prior to analysis.

### Meropenem Hydrolysis Assay (MHA)

MHA was performed on the reference and clinical strains after overnight growth on horse blood agar at 37°C in 5% CO_2_ atmosphere. Two different batches of meropenem trihydrate (Sigma-Aldrich, Milan, Italy) were received with six different distributions (5 arrived in ice and 1 at room temperature) and used during the study. A meropenem solution was obtained by resuspension in Tris-HCl 20 mM buffer (pH 6.8) at a concentration of 10 mM and immediately used after preparation or stored in aliquots at -20°C for 2–3 days before use without multiple refreezing. The 2,5-di-hydrossibenzoic acid (DHB) (Bruker Daltonics) diluted in TA30 solvent (30:70 v/v acetonitrile: 0.1% trifluoroacetic acid in water) was used as matrix. For each reference and clinical strain, a 4.0 McFarland (McF) bacterial suspension in 20 mM Tris-HCl, pH 6.8, was prepared. The MHA was performed using a protocol previously described [20] with some modifications. Briefly, 1 ml of each suspension was centrifuged at 14,000 x *g* for 6 min and the supernatant was removed. The pellet was re-suspended in 30 μl of 10 mM meropenem and 10 μl of 0.45% NaCl solution and air incubated for 2 h at 37°C in agitation. After the incubation, the suspension was centrifuged at 14,000 x *g* for 3 min and the supernatant was submitted to MALDI-TOF MS analysis. One microliter of the supernatant was mixed, in duplicate, with 1 μl of the DHB matrix directly on the wells of a polished steel MSP-96 target plate (Bruker Daltonics). The hydrolysis of meropenem was verified using the mass spectrometer MicroFlex LT (Bruker Daltonics) with the following parameter setting of the instrument: positive linear mode; laser frequency 64 Hz; ion source voltage 1, 20 kV; ion source voltage 2, 16.7 kV; target voltage, 7.0 kV; mass range, from 100 to 1200 Da; a total of 250 laser-shot, 50-shot for each step in different parts of the well on which had been deposited each sample, “laser” 66%, “detector gain” 6.6 and “resolution” 1.0. To improve the signal to noise ratio, at each laser-shot step the spectrum was considered only if the intensity of at least one of the expected peaks referring to intact (384, 406 and 428 Da) or hydrolyzed (358, 380, 402, and 424 Da) drug was >500 arbitrary units. The external calibrant standard Peptide Calibration Standard II (Bruker Daltonics) was employed for the calibration of the instrument in the 100 to 1200 Da range, according to the manufacturer's instructions. In each run, drug alone with 0.45% NaCl, the reference *K*. *pneumoniae* carbapenemase-producing strain (BAA-1705), as positive control, and the carbapenemase-nonproducing strain (BAA-1706), as negative control, were included. The obtained mass spectra were analysed by FlexAnalysis 3.3 software (Bruker Daltonics) in the range between 350 and 500 Da, and the m/z value and the intensity of each peak were displayed by MBT_Standard method.

When pure meropenem showed only peaks referring to intact drug, strains were classified as carbapenemase negative if only the peaks of meropenem (384 ±1 Da) and its sodium adducts (406 ±1 Da and 428 ±1 Da) were detected. Strains were classified as carbapenemase positive if the peaks for the hydrolyzed drug forms (358 ±1 Da, the decarboxylated product, 380 ±1 Da, 402 ±1 Da, and 424 ±1 Da, sodium salts of the decarboxylated product) appeared. If pure meropenem showed co-existence of peaks referring to both intact and hydrolyzed drug forms, the level of carbapenemase activity was calculated for each strain adapting the algorithm used by the automated MBT STAR-BL Software Module (Bruker Daltonics) as described by Papagiannitsis et al., 2015 [21] with some modifications. Briefly, this value was obtained by the difference between the log of the ratio of the summed hydrolyzed and intact meropenem intensities of each strain and that of the ratio of the meropenem alone tested in the same run. Strains with carbapenemase activity value (CAV) > 0.3 were interpreted as carbapenemase producers, whereas those with CAV ≤ 0.3 were interpreted as carbapenemase nonproducers.

## Results

**Fig 1:**
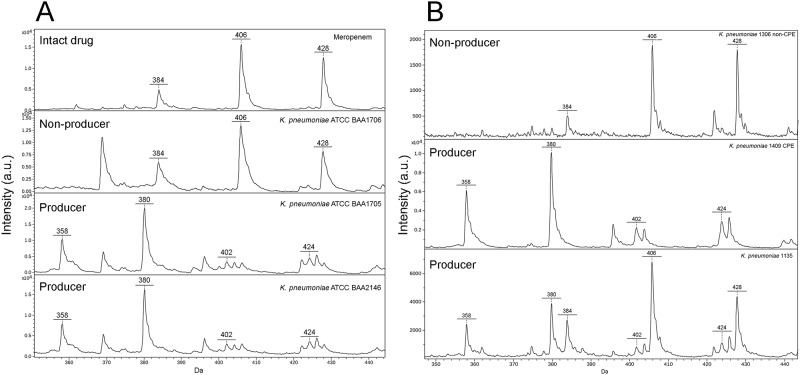
Spectra obtained by MHA. A) The spectrum obtained for the pure meropenem (arrived with ice) showed the peaks corresponding to the intact drug (384 ±1 Da) and to its sodium salts (406 ±1 Da; 428 ±1 Da); the spectrum obtained for the carbapenemase-nonproducing reference strain (*K*. *pneumoniae* ATCC BAA-1706) showed the same peaks as the pure meropenem, while the 2 carbapenemase-producing reference strains (*K*. *pneumoniae* ATCC BAA-1705, *bla*_*KPC*_ positive, and *K*. *pneumoniae* ATCC BAA-2146, *bla*_*NDM*_ positive) gave the peaks corresponding to the meropenem degradation (358 ±1 Da, the decarboxylated product) and to its sodium salts (380 ±1 Da; 402 ±1 Da; 424 ±1 Da). B) Example of spectra obtained for clinical samples tested with pure meropenem arrived with ice: a carbapenemase-nonproducing strain and two carbapenemase-producing strains, one of which (*K*. *pneumoniae* No. 1135 strain) showing the peaks corresponding to both the pure meropenem and the degradation products of the drug.

In all the experiments performed by the five drug distributions arrived with ice, the spectra obtained for the pure meropenem did not show peaks referring to degraded molecule and those obtained for the carbapenemase-nonproducing (ATCC BAA-1706) and carbapenemase-producing *K*. *pneumoniae* (ATCC BAA-1705, ATCC BAA-2146) reference strains showed the presence of the peaks corresponding only to the intact or to the hydrolyzed meropenem, respectively (Fig 1A). When the antibiotic received with the distribution delivered at room temperature was used, the spectra of meropenem alone as well as those obtained for the *K*. *pneumoniae* carbapenemase-nonproducing (ATCC BAA-1706), AmpC (NEQAS 2832 and NEQAS 3419), and ESBL (NEQAS 3253 and NEQAS 3341) reference strains showed the co-existence of the peaks of intact and degraded drug with CAV ≤ 0.17. On the contrary, the CAVs for the carbapenemase-producing reference strains (ATCC BAA-1705 and NCTC 13442) were ≥ 0.56.

All the 981 panel I strains were tested by MHA with intact drug and resulted CPE in agreement with the genotypical characterization (Fig 1B, Table 2). In particular, under this condition the peaks corresponding to meropenem degradation products appeared in 710 cases (537 KPC and 173 VIM) with complete disappearance of the intact form and in 271 cases (80 KPC and 191 VIM) with co-existence of both intact and hydrolysed forms (Fig 1B). Noteworthy, one of the genotypically characterized *bla*_*KPC*_ strains included in this panel (*K*. *pneumoniae* No. 1135 strain) was phenotypically identified as CPE only by MHA, whereas MHT and ST gave negative result (data not shown).

**Table 2:** Results of Meropenem Hydrolysis Assay (MHA) in case of intact or degraded drug in 1127 *Enterobacteriaceae* strains included in the panels I and II.

Panel	Carbapenemase type/resistance mechanism	Microorganism	No. of strains	MHA				
				Intact drug		Degraded drug		
				N°	Result	N°	CAV	Result
**I**			**981**	**981**	**Producers**	**0**	**NA**	**NA**
	KPC (617)	*K*. *pneumoniae*	603	603	Producers			
		*E*. *coli*	7	7	Producers			
		*E*. *aerogenes*	6	6	Producers			
		*M*. *morganii*	1	1	Producer			
	VIM (364)	*K*. *pneumoniae*	327	327	Producers			
		*E*. *cloacae*	17	17	Producers			
		*C*. *freundii*	12	12	Producers			
		*E*. *coli*	4	4	Producers			
		*E*. *aerogenes*	2	2	Producers			
		*K*. *oxytoca*	1	1	Producer			
		*S*. *marcescens*	1	1	Producer			
**II**			**146**	**2**	**Non producers**	**144**	**-0.36/0.25**	**Non producers**
	None (146)	*E*. *coli*	53			53	-0.30/0.24	Non producers
		*K*. *pneumoniae*	35	1	Non producer	34	-0.28/0.25	Non producers
		*E*. *cloacae*	13			13	-0.19/0.21	Non producers
		*K*. *oxytoca*	14	1	Non producer	13	-0.17/0.22	Non producers
		*P*. *mirabilis*	8			8	-0.14/0.13	Non producers
		*S*. *marcescens*	7			7	-0.18/0.20	Non producers
		*M*. *morganii*	5			5	-0.32/0.04	Non producers
		*C*. *koseri*	5			5	-0.21/0.15	Non producers
		*C*. *freundii*	3			3	-0.36/0.12	Non producers
		*E*. *aerogenes*	2			2	0.10/0.18	Non producers
		*P*. *stuartii*	1			1	-0.29	Non producer
**Total**			**1127**	**983**		**144**		

CAV: carbapenemase activity value;

NA: not applicable.

The 146 panel II strains (negative control group) tested by MHA resulted non-CPE as expected (Table 2). All the strains were tested with the degraded drug except two, which showed peak profiles comparable to that of pure meropenem without peaks referring to meropenem hydrolyzation (Fig 1B).

To further evaluate MHA on characterized strains other than those of the positive and negative control groups, MHA was applied on the 58 clinical strains of the third group containing carbapenemase enzyme types different from KPC and VIM, and resistance mechanisms other than carbapenemase production. In particular, 22 of these strains were tested by MHA with the intact drug and 36 with the degraded one. In 8 (1 NDM, 1 OXA-48 and 6 Class B carbapenemase strains other than NDM and VIM) out of the 22 strains tested with the intact drug, peaks corresponding to meropenem degradation products appeared; the remaining 14 strains (13 carbapenemase-nonproducing CRE and 1 ESBL) showed a peak profile comparable to that of pure meropenem without peaks referring to meropenem hydrolyzation. All the 36 strains (35 ESBL and 1 AmpC) tested with the degraded drug showed CAV under the cut-off. All results of MHA were in agreement with the resistance mechanism involved (Table 3).

**Table 3:** Results of Meropenem Hydrolysis Assay (MHA) in case of intact or degraded drug in 58 *Enterobacteriaceae* strains included in the panel III.

Carbapenem susceptibility	Carbapenemase type/resistance mechanism	Microorganism	No. of strains	MHA				
				Intact drug (n = 22)		Degraded drug (n = 36)		
				CNP	CP	CNP	CP	CAV
**Carbapenem nonsusceptible**			**21**	**13**	**8**	**0**	**0**	**NA**
	NDM (1)	*E*.*coli*	1		1			
	OXA-48 (1)	*K*.*pneumoniae*	1		1			
	Class B^ (6)	*K*. *pneumoniae*	5		5			
		*E*. *coli*	1		1			
	Unknown ^^(13)	*E*.*cloacae*	5	5				
		*K*. *pneumoniae*	4	4				
		*E*.*coli*	3	3				
		*E*. *aerogenes*	1	1				
**Carbapenem susceptible**			**37**	**1**	**0**	**36**	**0**	**-0.19/0.23**
	AmpC (1)	*E*. *cloacae*	1			1		-0.06
	ESBL (36)	*E*. *coli*	21	1		20		-0.15/0.15
		*K*. *pneumoniae*	10			10		-0.15/0.19
		*P*. *mirabilis*	3			3		-0.15/0.23
		*C*. *freundii*	2			2		-0.19/-0.05
**Total**			**58**	**14**	**8**	**36**	**0**	

CNP: carbapenemase nonproducer;

CP: carbapenemase producer;

CAV: carbapenemase activity value;

NA: not applicable;

**^** Class B carbapenemase strains other than NDM and VIM;

**^^** Carbapenemase-nonproducing enterobacteriaceae resistant to carbapenems.

**Fig 2:**
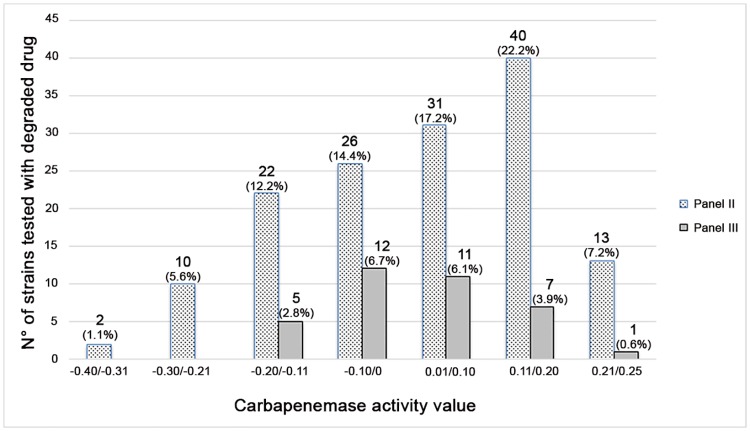
Frequency of the carbapenemase activity values of 180 strains analysed with the degraded drug. In brackets, the percentage of each frequency range calculated on the total of 180 strains are indicated.

The frequency of CAV of the overall 180 strains (144 from the negative control group and 36 from the third group) tested by MHA with the degraded molecule is shown in Fig 2.

## Discussion

Infections caused by CPE are now emerging worldwide and are difficult to treat [21]. Previous data have shown that strict epidemiological intervention based on the rapid detection of carbapenemase production can prevent the spread of those bacteria [21]. Therefore, the introduction and the evaluation of rapid and sensitive methodologies for the detection of carbapenemase-producing bacteria is of increasing importance. Phenotypic and DNA-based techniques are able to identify these carbapenemase producers, although with variable efficiencies [1]. In particular, MHT and ST are able to directly detect the carbapenemases but they need a time to results of at least 18h, are difficult to standardize and can give rise to false-positive results [11, 20]. On the other hand, the Carba NP test is recognized as a rapid assay but it suffers from a limited ability to detect OXA-type-producing isolates and some weak carbapenemases of class A, like GES-5 and SME-1 [21]. Conversely, PCR assays are rapid and highly sensitive and specific but, detecting only known enzyme-encoding genetic targets, they could be affected by the increasing number of types of carbapenemases [11, 22].

MALDI-TOF MS has been recently used to detect ß-lactamases, ESBLs and particularly carbapenemases [2, 23]. Several studies described its successful application for the detection of carbapenem hydrolysis (analysis of drug and their degradation products) in Gram negative rods using different drugs (mainly imipenem, ertapenem and meropenem), various reaction buffers (es. ammonium citrate, Tris-HCl, NH_4_HCO_3_), different matrixes (i.e. HCCA and DHB) and/or different incubation times [2, 11, 20, 21, 22, 24, 25]. The increasing introduction of this assay in the clinical microbiology laboratories, also directly from positive blood cultures [25], has led to the development of a software for the automatic interpretation of the results, which can be helpful for nonexperienced users facing difficulties with spectrum analysis [21].

In our study, a modified version of a previously described MHA [20], differing in meropenem concentration and in the matrix solvent, was applied on a large number of well-characterized EB strains, either carbapenemase producers or nonproducers.

In our experience, MHA demonstrated to be able to reveal the hydrolyzation of meropenem in all KPC or VIM carbapenemases, whereas no false positive results were obtained from fully susceptible *Enterobacteriaceae* strains. Interestingly, for a *bla*_*KPC*_ strain (*K*. *pneumoniae* No. 1135) non-CPE by MHT and ST, a partial hydrolysis of meropenem by MHA was observed after both 2h and 4h of incubation (data not shown). This is consistent with a low-expression of carbapenemase and it would have been missed by using only MHT and ST.

It is noteworthy that the specific peaks for meropenem did not completely disappear for the majority of the VIM carbapenemase-producing strains after 2h of incubation, when analyzed in presence of intact drug, despite a complete hydrolysis of meropenem after 30 min for the KPC-producing and after 1h for the class B (NDM and VIM) carbapenemase-producing reference strains was observed when the evaluation of different incubation times was performed (data not shown).

In this study, two different meropenem behaviours due to different shipment conditions were detected. When meropenem was delivered in ice, no degradation of drug alone was observed even after 4h of antibiotic incubation (data not shown), according also to other Authors [2, 20] and the spectra analysis was performed by the determination of the presence/absence of specific peaks. On the contrary, when meropenem was delivered without ice, the drug alone showed degradation, as reported in the literature for other carbapenems [11, 24]. Since this phenomenon could mislead the result’s interpretation, the spectra analysis performed could not be merely based on the presence/absence of specific peaks but it needed the calculation of CAV to distinguish between carbapenemase-producing and -nonproducing strains. Although an automatic software (MBT STAR-BL Module Software, Bruker Daltonics) is available to determine the level of β-lactamase activity (normalised logRQ), in this study the CAV was evaluated by a formula adapted from that used by this module [21]. Taking into account that this software has an additional cost, the manual application of the formula can be widely extended to clinical laboratories without further charges and instrument updates with reliable results, as demonstrated in this study.

MBT STAR-BL Software indicates as carbapenemase producers or nonproducers those strains with a normalised logRQ value above to 0.4 or below to 0.2, respectively. A normalised logRQ value comprised between 0.2 and 0.4 indicates an ambiguous hydrolysis measurement that requires re-testing or prolonging the incubation time [25]. Considering that our formula not completely overlaps that proposed by the manufacturer, in this study a cut-off for CAV was set at 0.3 on the basis of the results obtained from Panel II (91%, 131/144, with CAVs below 0.20 and only 9%, 13/144, with CAVs ranging from 0.21 to 0.25) and from carbapenemase-producing reference strains (CAVs ≥ 0.56).

Despite on a limited number of strains, MHA has proved to be able to successfully distinguish between carbapenemase-producing and -nonproducing *Enterobacteriaceae* when applied also to NDM and OXA-48-producing strains and to CRE with resistance mechanisms other than carbapenemase production, as well as to AmpC and ESBL strains.

Finally, MHA by MALDI-TOF MS analysis is independent of the type of carbapenemases involved, and it is faster (2h *vs*. 18h) and easier to perform/interpret than culture-based methods. Moreover, the reagent cost per determination is low (less than 1 Euro). Undoubtedly, the instrument cost would significantly increase the MHA expense; however, in the constantly increasing microbiology laboratories using the MALDI-TOF mass spectrometer for the routine identification of microbial strains [11, 22], its cost is spread on the different activities.

Taking into account all these considerations, the proposed version of MHA could be helpful to rapidly provide fundamental information for the clinical management of the patient and for the control of the spread of infections by CPE.

## Supporting information

S1 Text Description of the isolation from clinical samples, of the identification and antimicrobial susceptibility testing procedures. (DOCX)

S2 Text Procedures for the detection of carbapenemase genes. (DOCX)
