# Supplementary material for: Evaluation of a modified meropenem hydrolysis assay on a large cohort of KPC and VIM carbapenemase-producing Enterobacteriaceae
Source: PLoS One. 2017 Apr 6;12(4):e0174908. doi: 10.1371/journal.pone.0174908 (PMC5383100; doi:10.1371/journal.pone.0174908)
Supplement: S1 Text — (DOCX) [file pone.0174908.s001.docx]

**Description of isolation from clinical samples, identification and antimicrobial susceptibility testing procedures.**

**The rectal swabs (509) were collected from patients admitted to the intensive care units of the hospital** as part of active surveillance of CPE according to the recommendations of both the Italian Ministry of Health and the Emilia-Romagna region [1, 2]**. The rectal swabs were inoculated on chromogenic agar (Brilliance CRE Agar; Oxoid, Milan, Italy) and the blue colonies referring to presumptive CRE belonging to the KESC group (*Klebsiella, Enterobacter, Serratia, Citrobacter*), as reported by the manufacturer, were subcultured on MacConkey agar (Kima, Piove di Sacco, Italy) with a 10 µg disk of ertapenem (Oxoid) in order to confirm the resistance to carbapenems. The strains nonsusceptible to ertapenem were identified at species level by MALDI-TOF MS (Bruker Daltonics, Bremen, Germany) and submitted to the antimicrobial susceptibility testing (AST) with the BD-Phoenix system using the Gram-Negative NMIC/ID88 or NMIC/ID94 Combo Panels (Beckton Dickinson, Sparks, MD, USA) including the biochemical identification in order to confirm the purity of each strain, according to the manufacturer's instructions. AST was interpreted according to MIC breakpoint criteria of** the **CLSI on 2014 [3].**

The strains from the clinical samples other than rectal swabs (676) were isolated on blood agar (Kima) and McConkey agar after direct inoculum according to standard procedures [4] or after subculture from Bactec blood culture bottles (Becton Dickinson) previously inoculated with normally sterile fluids [5]. The isolated strains were identified and subjected to AST as described above.

References.

1. Ministero della salute. Available: http://www.trovanorme.salute.gov.it/norme/renderNormsanPdf;jsessionid=MAw15Ht7RIvegw+S4afxWA.sgc4-prd-sal?anno=0&codLeg=45499&parte=1%20&serie= [Accessed 30 November 2015].
2. Gagliotti C, Cappelli V, Carretto E, Pan A, Sarti M, Suzzi R et al. 2013. Indicazioni pratiche e protocolli operativi per la diagnosi, la sorveglianza e il controllo degli enterobatteri produttori di carbapenemasi nelle strutture sanitarie e socio-sanitarie. Available:

http://assr.regione.emilia-romagna.it/it/servizi/Indice_A...Z/C/carbapenemasi. [Accessed 23 July 2015].

1. Clinical and Laboratory Standards Institute. Performance standards for antimicrobial susceptibility testing; twenty-fourth informational supplement. CLSI document M100-S24. Wayne, PA: Clinical and Laboratory Standards Institute, 2014.
2. Isenberg HD. Clinical Microbiology Procedures Handbook. 2^nd^ Edition update. Washington, D.C.; ASM Press, 2007.
3. Calderaro A, Martinelli M, Motta F, Larini S, Arcangeletti MC, Medici MC, et al. Comparison of peptide nucleic acid fluorescence *in situ* hybridization assays with culture-based matrix-assisted laser desorption/ ionization-time of flight mass spectrometry for the identification of bacteria and yeasts from blood cultures and cerebrospinal fluid cultures. Clin Microbiol Infect. 2014;20: O468-75.
