# Supplementary material for: Evaluation of a modified meropenem hydrolysis assay on a large cohort of KPC and VIM carbapenemase-producing Enterobacteriaceae
Source: PLoS One. 2017 Apr 6;12(4):e0174908. doi: 10.1371/journal.pone.0174908 (PMC5383100; doi:10.1371/journal.pone.0174908)
Supplement: S2 Text — (DOCX) [file pone.0174908.s002.docx]

**Procedures for the detection of carbapenemase genes.**

To detect carbapenemase genes on CRE strains grown overnight on MacConkey agar at 37° C in 5% CO_2_ atmosphere 2 different molecular assays were used: 676 strains were characterized by different single Real-Time PCRs and 326 by “Check-Direct CPE” assay for BD MAX.

A 5 McF bacterial suspension in saline buffer (NaCl 0.85% Medium; bioMérieux, Marcy L'Etoile, France) of each of the 676 strains was submitted to DNA extraction using the automatized MAgNA Pure LC by the "MagNA Pure LC DNA Isolation Kit III" (Roche Diagnostics, Mannheim, Germany), according to the manufacturer’s instructions. On the basis of carbapenemase class obtained by ST, DNA was used to perform 4 single Real-Time PCR (RT-PCR) assays targeting *bla_KPC_*, *bla_NDM_* and 16S rRNA (internal control), respectively [1] and *bla_VIM_* [2], with some modifications. Briefly, each run was performed in an ABI PRISM 7300 Real-Time PCR system (SDS version 1.3.1) instrument (Applied Biosystems, Foster City, CA, USA) by using a TaqMan 2x Universal MasterMix (Applied Biosystems), including a negative extraction control, a negative PCR control, and a positive control for each target (ATCC BAA-1705 for *bla_KPC_*_,_ ATCC BAA-2146 for *bla_NDM_*, a well-characterized *K. pneumoniae* collection strain for *bla_VIM_*, and ATCC BAA-1706 for 16sRNA) (Table 1).

To perform the genotypic characterization by the “Check-Direct CPE” assay for BD MAX, a bacterial suspension (0.5-1.0 McF) in saline buffer of each of the 326 strains was submitted to DNA extraction and to real-time amplification, both performed by the BD MAX system according to the manufacturer’s instructions. Each run included one negative PCR control (double-distilled sterile water), one negative extraction control (saline buffer), and one positive PCR control (included into the assay kit).

1. Centers for Disease Control and Prevention. Available:

http://www.cdc.gov/HAI/pdfs/labSettings/KPC-NDM-protocol-2011.pdf [Accessed 23 July 2015].

1. van der Zee A, Roorda L, Bosman G, Fluit AC, Hermans M, Smits PH et al. Multi-centre evaluation of real-time multiplex PCR for detection of carbapenemase genes OXA-48, VIM, IMP, NDM and KPC. BMC Infect Dis. 2014;14: 27.

**Table 1. Primers, probes and** t**hermal cycling conditions used in this study.**

| **Gene** | **Probe and Primers** | **Sequence** |
| --- | --- | --- |
| **KPC** | KPC-Probe  KPC-F  KPC-R | 5’ FAM-TGATAACGCCGCCGCCAATTTGT-BHQ1 3’  5’ GGCCGCCGTGCAATAC 3’  5’ GCCGCCCAACTCCTTCA 3’ |
| **NDM** | NDM-Probe  NDM-F  NDM-R | 5’ Yakima Yellow-TGGATCAAGCAGGAGAT-BHQ1 3’  5’ GACCGCCCAGATCCTCAA 3’  5’ CGCGACCGGCAGGTT 3’ |
| **16S rRNA** | 16S rRNA  16S rRNA F  16S rRNA R | 5’ Dragonfly Orange- CACGAGCTGACGACAR*CCATGCA-BHQ2 3’  5’ TGGAGCATGTGGTTTAATTCGA 3’  5’ TGCGGGACTTAACCCAACA 3’ |

* R = A or G

| Reagent (for each target) | | **Volume/Concentration** |  |  |
| --- | --- | --- | --- | --- |
| Taqman 2x Universal Mastermix | | 10 µl | | |
| Primer Forward | | 0.5 µM | | |
| Primer Reverse | | 0.5 µM | | |
| Probe | | 0.25 µM | | |
| DNA | | 2 µl | | |

Mix: 20 µl

Thermal cycling conditions are as follows: 
1) Enzyme activation step: 95°C for 3 minutes; 
2) 40 PCR cycles of : 95°C for 3 seconds, 60°C for 30 seconds.

According to reference 1.

| Gene | **Probe and Primers** | **Sequence** |
| --- | --- | --- |
| **VIM** | VIM-Probe (FAM)  VIM-F  VIM-R | 5’ FAM- ACGCAGTGCGCTTCGGTCCAGT –BHQ1 3’  5’ GAGATTCCCACGCA[C/T]TCTCTAGA 3’  5’ AATGCGCAGCACCAGGATAG 3’ |

| Reagent | | **Volume/Concentration** |  |  |
| --- | --- | --- | --- | --- |
| Taqman 2x Universal Mastermix | | 12.5 µl | | |
| Primer Forward | | 0.6 µM | | |
| Primer Reverse | | 0.6 µM | | |
| Probe | | 0.14 µM | | |
| DNA | | 2 µl | | |

Mix: 25 µl

Thermal cycling conditions are as follows: 
1) Enzyme activation step: 95°C for 3 minutes;
2) 40 PCR cycles of : 94°C for 15 seconds, 60°C for 1 minute.

According to reference 2.
